# Machine Learning for Predicting Chemical Potentials of Multifunctional Organic Compounds in Atmospherically Relevant Solutions

**DOI:** 10.1021/acs.jpclett.2c02612

**Published:** 2022-10-19

**Authors:** Noora Hyttinen, Antti Pihlajamäki, Hannu Häkkinen

**Affiliations:** † Department of Chemistry, Nanoscience Center, University of Jyväskylä, FI-40014 Jyväskylä, Finland; ‡ Department of Physics, Nanoscience Center, University of Jyväskylä, FI-40014 Jyväskylä, Finland

## Abstract

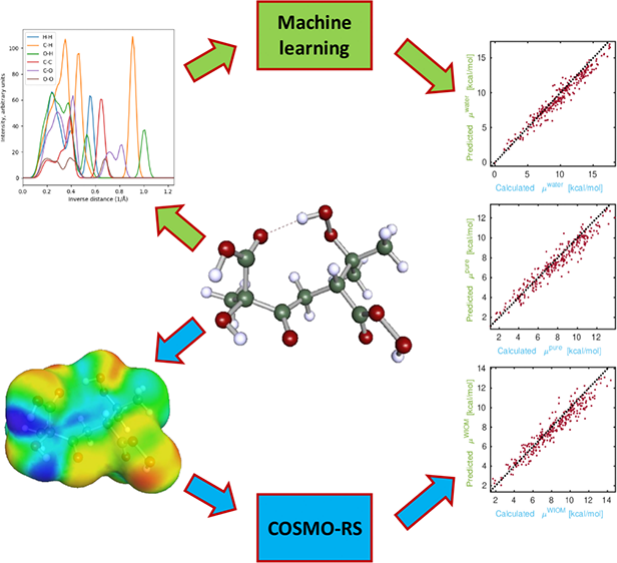

We have trained the Extreme Minimum Learning Machine (EMLM) machine learning model to predict chemical potentials of individual conformers of multifunctional organic compounds containing carbon, hydrogen, and oxygen. The model is able to predict chemical potentials of molecules that are in the size range of the training data with a root-mean-square error (RMSE) of 0.5 kcal/mol. There is also a linear correlation between calculated and predicted chemical potentials of molecules that are larger than those included in the training set. Finding the lowest chemical potential conformers is useful in condensed phase thermodynamic property calculations, in order to reduce the number of computationally demanding density functional theory calculations.

Condensed-phase thermodynamic properties are important in the modeling of the formation and growth of atmospheric aerosols. In recent years, thermodynamic properties, such as saturation vapor pressures and activity coefficients, have been calculated using the Conductor-like Screening Model for Real Solvents (COSMO-RS^1−3^ implemented, e.g., in the COSMO*therm* program^4^).^5−14^ As input, the COSMO-RS model uses single molecule density functional theory (DFT) results of multiple conformers for statistical thermodynamics calculations. The advantage of COSMO-RS is that, unlike group-contribution methods (e.g., AIOMFAC,^15^ SIMPOL.1^16^), intramolecular interactions between functional groups are included in the model by including different conformers of each molecule. Additionally, the COSMO-RS model does not need to be parametrized for new types of compounds.

A large uncertainty in COSMO*therm* calculations originates from the selection of conformers for the calculations.^8,17^ Especially multifunctional compounds can have various intramolecular hydrogen bonding patterns and generally all conformers cannot be included in the COSMO*therm* calculations due to memory limitations. The hydrogen bond acceptors and donors available for intermolecular hydrogen bonding determine how strongly the compound is able to interact with the surrounding system. We have therefore used the number of intramolecular H-bonds to select conformers for COSMO*therm* calculations in previous studies.^8,11−13,17−19^ There is a strong correlation between the number of intramolecular H-bonds and the chemical potential, which is used in COSMO-RS to describe the interaction between a compound and the surrounding system (see Figure S1 of the Supporting Information). For example, in polar solutions such as water, conformers containing no intramolecular H-bonds are able to interact with the surrounding system, leading to relatively low chemical potentials. On the other hand, conformers containing multiple intramolecular H-bonds may be more favorable in nonpolar systems. In order to find all relevant conformers, the whole conformational space needs to be sampled. However, this method requires quantum chemical calculations on a large number of conformers and becomes computationally expensive, when the number of possible conformers increases exponentially with the torsional degrees of freedom of a molecule.

Here, we utilize a distance-based machine learning (ML) method to improve the conformer selection process. Kernel Ridge Regression (KRR) methods have been used recently in atmospheric science to predict binding energies of small clusters^20^ and saturation vapor pressures of atmospherically relevant organic compounds.^21^ We have chosen to use a distance-based ridge regression model Extreme Minimal Learning Machine (EMLM^22^), which was recently used to predict energies of thiolate protected gold nanocluster conformers.^23^ EMLM is a computationally light ML method. Additionally, it has only a single hyperparameter, the number of reference points. Hence, it does not require tedious hyperparameter optimization. This is a significant advantage, because the descriptors of the atomic structures often contain several parameters to be tested. In order to find suitable conformer distributions for different atmospherically relevant systems (aqueous, organic), the model was trained to predict condensed-phase chemical potentials of different conformers of atmospherically relevant multifunctional organic compounds. The elemental composition and geometry of the conformers were encoded for the ML model using a global descriptor called many-body tensor representation (MBTR^24^), implemented in DScribe.^25^

To train and test the model, we used atmospherically relevant multifunctional compounds generated with the Generator of Explicit Chemistry and Kinetics of Organics in the Atmosphere (GECKO-A^26,27^). GECKO-A is a data processing tool that generates gas-phase oxidation products in tropospheric conditions. For our study, we selected only those compounds that were flagged as products of α-pinene oxidation and that contain only carbon, oxygen, and hydrogen atoms (excluding nitrogen containing compounds) by Isaacman-VanWertz and Aumont,^28^ 2283 molecules in total. These compounds contain hydroxyl, carbonyl, carboxylic acid, hydroperoxide, peroxy acid, and peroxide functional groups. 284 of the molecules were separated for testing, and the remaining 1999 molecules were used in the training of the EMLM model. In order to include a good variation of different conformers and molecules into the model, the training data molecules from Isaacman-VanWertz and Aumont^28^ were divided into 2 different types of training data differing in number of conformers and geometry optimization method. In the first training set (labeled as train1), 50 conformers were generated for 1800 molecules using the Merck molecular force field (MMFF^29^) in the Balloon program.^30^ The MMFF94 parametrization in Balloon was edited to include peroxy acid groups (see Section S1 of the Supporting Information). In the second training set (labeled as train2), we found all conformers of the remaining 199 molecules using a systematic conformer sampling algorithm of the Spartan program^31^ and the geometries were optimized at the BP/def-TZVP level of theory using the TURBOMOLE program package.^32^ Duplicate conformers were omitted after the geometry optimization using the CLUSTER_GEOCHECK algorithm of the COSMO*conf* program.^33^ Using geometries optimized at different levels of theory helps to account for the small differences in bond distances and angles between the methods. As a third training set (labeled as train3), we used a small set (2956 conformers of 125 molecules) of COSMO files generated for potential α-pinene-derived SOA constituents.^34^ These conformers were obtained with similar conformer sampling and geometry optimization methods as train2, but the data set contains some larger molecules than those included in train1 and train2. All three training sets were used to train one EMLM model, 155 867 structures in total. The chemical potentials (pseudochemical potential, see Section S2 of the Supporting Information) at 298.15 K were calculated using single-point BP/def2-TZVPD-FINE COSMO calculations and the BP_TZVPD_FINE_21 parametrization of COSMO*therm*.

The performance of the EMLM model was tested using 3 data sets:

Test1: 284 molecules from the Isaacman-VanWertz and Aumont^28^ molecule set, not included in the training of the model. The conformers in test1 and train1 were generated similarly, with 50 conformers for each molecule.
Test2: All found conformers (2841) of a single molecule (CC(O)(C(=O)(O))C(=O)CC(C(=O)(OO))C(OO)(C)C), found using the systematic conformer sampling of Spartan. The geometries were optimized at the BP/def-TZVP level of theory. 50 conformers of this molecule (optimized using the MMFF force field) were already included in the training data. This molecule is among the largest molecules in the GECKO-A data set and contains most atmospherically relevant functional group types (hydroxy, ketone, hydroperoxy, carboxylic acid, and peroxy acid).
Test3: 15 accretion products (dimers) from the α-pinene + OH reaction (C_20_H_34_O_10_),^35^ optimized at the BP/def-TZVP level of theory (11 496 conformers). This data set is testing the performance of the model when extrapolating to larger molecules outside the training data of the model.

The distributions of carbon and oxygen number of the molecules in the training and test data are shown in Figures 1a and 1b, respectively. Test2 and test3 are not shown in Figure 1 because they contain only conformers of one elemental composition (C_10_H_16_O_9_ and C_20_H_30_O_10_, respectively).

**Figure 1:**
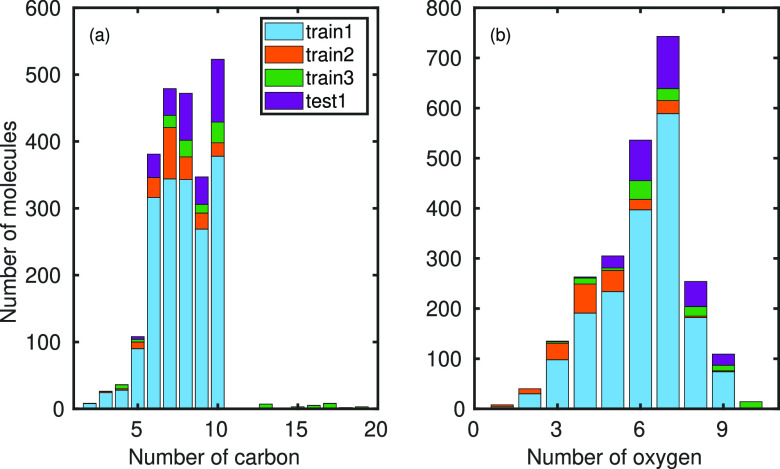
Numbers of carbon and oxygen atoms in the molecules included in the training and test data sets. Note that the number of conformers for each molecule is much larger in train2 (62 911 conformers in total for 199 molecules) compared to train1 and test1 (50 conformers of each molecule).

We visualized the MBTR descriptors using Principal Component Analysis.^36^Figure 2a and 2b show how the first and second principal components (PC1 and PC2, respectively) correlate with the chemical potential in infinite dilution in water. We see that, in terms of the PC1 values, test3 (C_20_H_30_O_10_) is clearly different from the other data sets. Similarly, some of the conformers of the train3 have relative high PC1 values. This principal component is most likely reflective of the size of the molecule, since these two data sets (train3 and test3) include molecules that have significantly higher molar masses than the other 4 data sets. The differences in the PC1 of test3 are likely caused by the different functional groups of the C_20_H_30_O_10_ isomers. On the other hand, test2 (C_10_H_16_O_9_) has almost identical PC1 values for all the data points, because the data set includes conformers of a single isomer. Additionally, we can see no correlation between the first principal component and chemical potential. Similarly, there is no correlation between PC2 and chemical potential in water (Figure 2b). We can see that, other than test3, our testing data are in the same feature space with our training data. There is also no visible separation between the force field (1) and DFT optimized (2 and 3) data sets.

**Figure 2:**
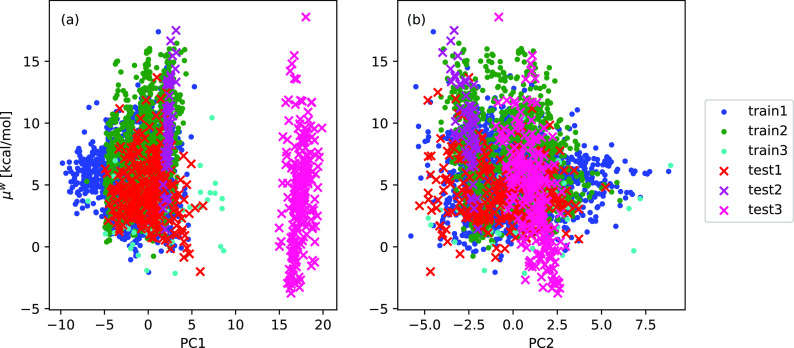
Correlation between chemical potential in water (μ^w^) and (a) PC1 and (b) PC2 from the principal component analysis (PCA) of the MBTR descriptors of the different training and test data sets. For clarity, only 2% of the data points are shown in the figure.

A common way to predict the potential energy of atomic systems is to use ML methods that estimate the energy as a sum of local contributions. However, due to the fact that chemical potential does not depend on the size of the compound, a summation approach would require extra scaling. Our global ML model does not have this characteristic making it a viable option for chemical potential predictions.

Figure 3 shows the correlation between calculated and EMLM-predicted chemical potentials of 3 different test data sets in water. Note that only 2%, 10%, and 20% of the data points are shown in Figure 3a, 3b, and 3c (2 of the 15 isomers), respectively. Similar figures for pure compound and infinite dilution in water-insoluble organic matter (WIOM, CC(=O)C1OC(C)(OC(C2=CC(C)=CC(C)=C2)O3)C3O1^37^) are shown in Figures S4 and S5 of the Supporting Information, respectively. The test1 data set contains one outlier conformer with predicted chemical potential hundreds of kcal/mol outside the range of any calculated chemical potentials. The large error in the prediction was caused by an unrealistic bond angle in the conformer, and the conformer was therefore omitted from the analysis.

**Figure 3:**
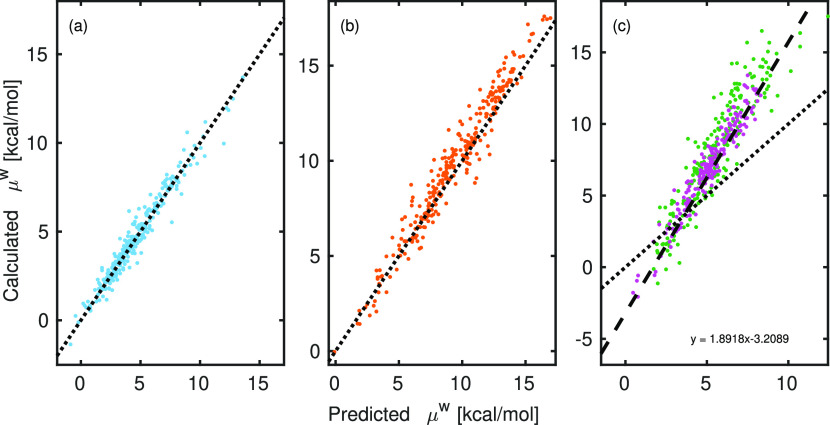
Predicted and calculated chemical potentials (μ^w^) of (a) test1, (b) test2, and (c) test3 in water solvent. Only a small subset of the test data points, taken at constant intervals, are shown in the figures for clarity. In (c), the dashed line is a linear fit to all of the molecules in test3. The green and magenta points are for the isomers with the highest and lowest RMSE between the calculated values and predicted values scaled with the linear fit.

Our model is able to predict the chemical potentials of test1 and test2 data sets very well in all three solvents. For test3 (C_20_H_30_O_10_), the RMSE of the predicted chemical potentials in all solvents are significantly larger than for the other test data (smaller molecules). This is caused by not having included molecules with similar sizes to the training data and the ML model is extrapolating outside its training region. From Figure 3c we see that even though the correlation between predicted and calculated chemical potentials is good, the model is not able to predict the absolute values of the chemical potentials. We therefore fit a line to the test3 data points and calculated the RMSE after scaling the predicted chemical potentials with the fitted equation. The RMSE of all test data sets (scaled RMSE for test3) in the three solvents are shown in Table 1.

**Table 1:** Root Means Square Errors (RMSE) of the Test Data Sets for Chemical Potentials in Different Solvents in kcal/mol

	water	pure	WIOM
test1	0.53	0.46	0.45
test2	0.87	0.73	0.74
test3^a^	1.37	1.17	1.17

a The RMSE of test3 was calculated by first scaling the predicted chemical potentials with a linear fit to all points of test3.

The prediction is the most accurate for test1, around 0.5 kcal/mol. For the molecule of test2, there is a smaller representation of similar molecules in the training data, because it is one of the largest molecules in the GECKO-A data set with 9 oxygen atoms, which is seen in Figure 2. On the other hand, test1 is more evenly spread in the principal component space. Even though test3 is well outside the size range of the training data, there is a linear correlation between the EMLM-predicted and calculated chemical potentials. Using a different subset of the Isaacman-VanWertz and Aumon^28^ data set as test1 leads to similar results: 0.56, 0.81, and 1.34 kcal/mol RMSE for the chemical potential in infinite dilution in water for test1, test2, and test3, respectively.

We further tested how well EMLM can predict chemical potentials of molecules that are larger than the molecules included in the training data (see Section S3 of the Supporting Information). The EMLM model is able to predict chemical potentials of molecules containing up to 4 more non-hydrogen atoms than the molecules of the training data set with good accuracy. The prediction deteriorates quickly when the size of the predicted molecules is increased. We were not able to discern any difference in the prediction accuracy based on functional groups in the molecule. For example, the highest and lowest RMSE values among the molecules containing 9 non-hydrogen atoms more than the training data (3.8 and 0.9 kcal/mol, respectively) both had identical functional groups (3 carbonyl and 3 hydroperoxide).

Here, we chose the 3 systems (water, pure compound, and WIOM) for their atmospheric relevance. The error is very close to equal in the prediction of chemical potentials in pure compound and in WIOM, and smaller than in water for all of the test data sets. Since the MBTR descriptors, as well as the screening charge densities used to calculate the target chemical potentials, are identical in all of the models, there may be some additional uncertainty arising from the COSMO*therm* calculation of chemical potential in infinite dilution in water. Alternatively, there may be some features critical for the calculation of chemical potential in water but not in pure compound or WIOM, which are not captured by the MBTR descriptor.

Our model is optimal for applications that require a set of low chemical potential conformers, as opposed to accurate absolute chemical potentials. In its current form, the model includes carbon, hydrogen, and oxygen atoms. The model can be extended to include other atoms by adding, e.g., nitrogen-containing molecules to the training data. Lastly, we give example codes for using chemical potential predictions in finding conformers for COSMO*therm* calculations (see the Supporting Information). The EMLM chemical potential prediction can be added to a COSMO*conf* calculation routine between conformer sampling and the first DFT calculations. After the chemical potential prediction, high chemical potential conformers can be discarded from the calculation. A large fraction of the conformers are often classified as duplicates after the DFT optimization based on geometries and similarity of chemical potentials in a set of solvents. The number of conformers kept after the chemical potential prediction should therefore be sufficiently high in order to ensure that enough conformers remain after all steps of the COSMO*conf* calculation.

In conclusion, we have shown that machine learning can be used to predict chemical potentials of individual conformers of atmospherically relevant multifunctional organic compounds. The chemical potentials can be used to find more realistic condensed-phase conformer distributions for COSMO*therm* calculations, increasing the reliability of thermodynamic property estimates. COSMO*therm* has an enormous potential for estimating thermodynamic properties of atmospheric multifunctional compounds, whose properties are experimentally out of reach, and our computationally cheaper calculation method for selecting conformers will allow for the inclusion of a larger number of compounds with different thermodynamic properties to atmospheric aerosol models. Additionally, the lowest chemical potential conformers can be used to parametrize new COSMO-RS implementations, such as the new open source openCOSMO-RS.^38^

## Methods

The equations used for creating the Many-Body Tensor Representation (MBTR) are described in Section S4 of the Supporting Information. The lowest root-mean-square error (RMSE) between predicted and calculated chemical potentials in the infinite dilution in water was found by including *k* = 1 (atomic numbers), *k* = 2 (atom distances), and *k* = 3 (angles between atoms) in the MBTR descriptor. We optimized two adjustable parameters of the distance and angle tensors: σ and the scaling factor *s*. Higher σ values (broadening) mean that slightly different atom distances (e.g., from different geometry optimization methods) fit under the same peak, while lower σ values highlight even small differences between the conformers. The scaling factor determines the exponential weighting of the functions based on the atom distances so that higher values of *s* give less weight to atom pairs that are farther apart.

The MBTR parameters were optimized using a small fraction of the whole data (1%), in order to conserve time and memory in the generation of the MBTR files. First, 10% of all conformers were selected at constant intervals (every 10th conformer based on the conformer numbering of the conformer sampling program) from the whole data sets. Subsequently, 10% of those conformers were selected by the Euclidean distances of their MBTR descriptors using the RS-maximin algorithm described by Gonzalez^39^ and Hämäläinen et al.^40^ In short, the data point closest to the mean of all data points is selected as the first reference point and all following reference points are selected so that their distance to the already selected points is maximized.

The final MBTR parameters selected for the model are σ = 0.025 and *s* = 0.5 for *k* = 2 (distance tensors) and σ = 0.12 and *s* = 0.8 for *k* = 3 (angle tensors). The *k* = 1 tensors were included with σ = 0.04. If *k* = 3 is left out of the descriptor, the RMSE is around 55% higher (10% for dimers) than when *k* = 3 is included (see Figure S6 of the Supporting Information). It is possible to use the model containing only *k* = 1 and *k* = 2 to decrease the calculation time, because including *k* = 3 increases the size of the MBTR descriptor by 365%. Excluding the *k* = 3 tensor is recommended especially if more atoms are added to the current three-atom model.

The Extreme Minimal Learning Machine (EMLM) is described in detail in Section S5 of the Supporting Information. In our calculations, all values in the training data descriptor were minmax scaled between 0 and 1, and the chemical potentials were minmax scaled between −1 and 1. In the final model, 25% of the whole training data were used as reference points, selected using the RS-maximin algorithm (see Figure S9 of the Supporting Information).

## Supporting Information Available

Section S1: Comparison of MMFF and BP geometries, Figure S3: Correlation between H-bonds and chemical potential, Figures S4 and S5: chemical potential predictions in pure compound and WIOM, Section S2: ML Model Extrapolation, Section S3: Many-Body Tensor Representation, Section S4: Extreme Minimal Learning Machine, Figure S9: Model convergence (PDF)

## Notes

The cosmo-files used to train and test the model (excluding the previously published train3 and test3), and codes for predicting chemical potentials can be accessed through Jyväskylä University Digital Repository (JYX) at: https://doi.org/10.17011/jyx/dataset/83604.
