# Supplementary material for: Machine Learning for Predicting Chemical Potentials of Multifunctional Organic Compounds in Atmospherically Relevant Solutions
Source: J Phys Chem Lett. 2022 Oct 19;13(42):9928–33. doi: 10.1021/acs.jpclett.2c02612 (PMC9619930; doi:10.1021/acs.jpclett.2c02612)
Supplement: Supplementary file 1 — jz2c02612_si_001.pdf [file jz2c02612_si_001.pdf]

Supporting Information: Machine Learning for Predicting Chemical Potentials of Multifunctional Organic Compounds in Atmospherically Relevant Solutions

Noora Hyttinen,[*,†] Antti Pihlajamäki,[‡] and Hannu Häkkinen[†,‡]

†*Department of Chemistry, Nanoscience Center, University of Jyväskylä, FI-40014 Jyväskylä, Finland*

‡*Department of Physics, Nanoscience Center, University of Jyväskylä, FI-40014 Jyväskylä, Finland*

E-mail: noora.x.hyttinen@jyu.fi

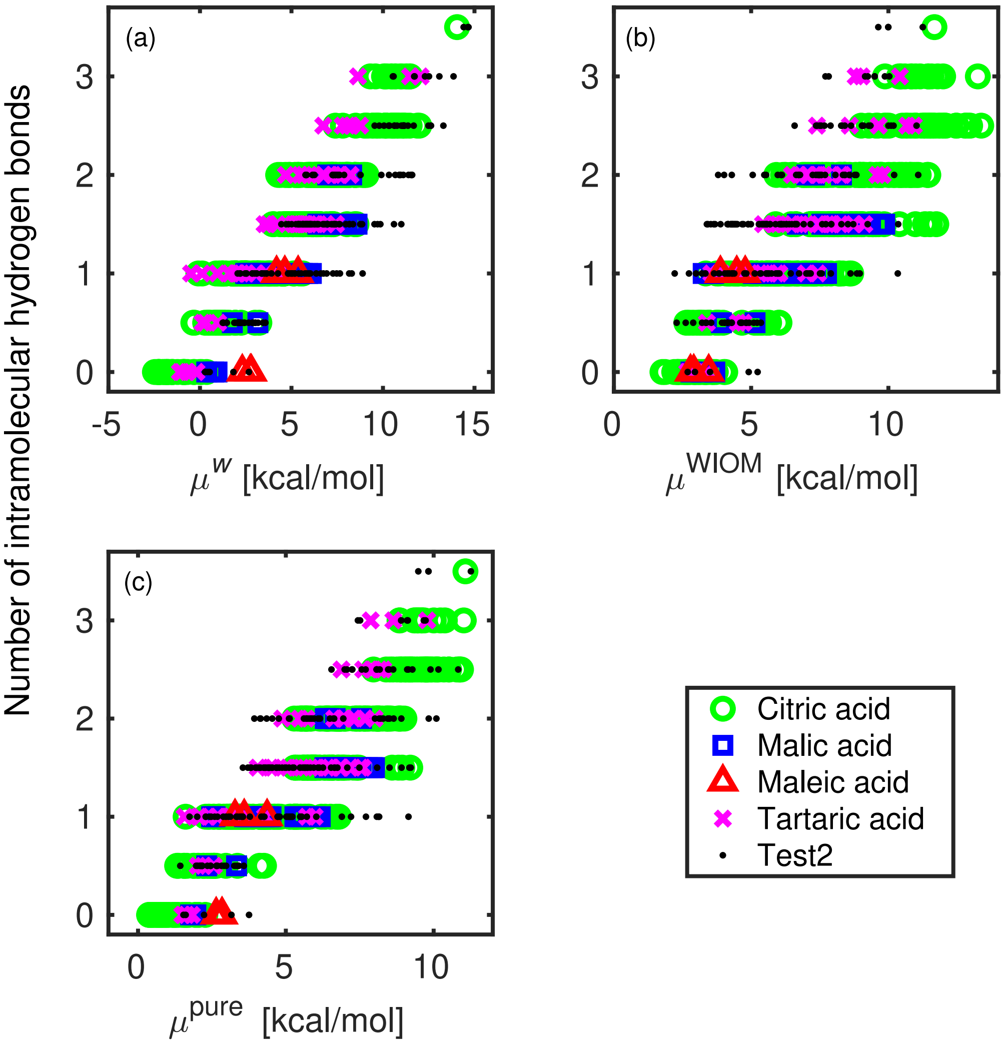

Figure S1: Correlation between the number of intramolecular hydrogen bonds and the chemical potential (μ) in infinite dilution in (a) water, (b) WIOM and (c) pure compound at 298.15 K. Partial hydrogen bonds (classified by COSMO*therm*) are included here as 0.5 H-bonds to the number of intramolecular hydrogen bonds. COSMO files of the acids were taken from Hyttinen and Prisle.[S1]

S1 Comparison of MMFF and BP/TZVP geometries

In order to improve the geometries obtained from conformer sampling, we made small changes to the existing MMFF94 force field. The MMFF94 force field does not include a parameter for peroxy acid, which leads to geometries where the hydrogen of the peroxy acid group is not on the same plane with the other atoms of the group. However, geometries optimized using density functional theory (DFT, e.g., BP/def-TZVP), have all atoms of a peroxy acid in one plane, unless the hydrogen of the peroxy acid group forms an intramolecular hydrogen bond with another H-bond acceptor of the molecule. We added a parameter that forces the hydrogen to the same plane, unless it is interacting with other H-bond acceptors of the molecule.

In order to find the effect of DFT geometry optimization on COSMO energy and chemical potential we ran a small set of test calculations. First, we generated conformers using the Balloon program[S2] with 120 generations keeping 100 conformers. Both MMFF94 and the edited MMFF force fields were used in separate conformer searches. Single-point COSMO calculations of these geometries were performed at the BP/def2-TZVPD-FINE level of theory using TURBOMOLE.[S3] Additionally, the geometries of both sets of conformers were optimized at the BP/def-TZVP level of theory, with BP/def2-TZVPD-FINE single-point energy calculations. Each of the conformer sets represents a small sample of all possible conformers of the molecule of the test2 data set (CC(O)(C(=O)(O))C(=O)CC(C(=O)(OO))C(OO)(C)C). Figure S2 shows how the chemical potential in water and COSMO energy change between the MMFF and BP/def-TZVP geometry optimizations. The chemical potentials were calculated using the BP_TZVPD_FINE_21 parametrization of COSMO*therm*2021.[S4]

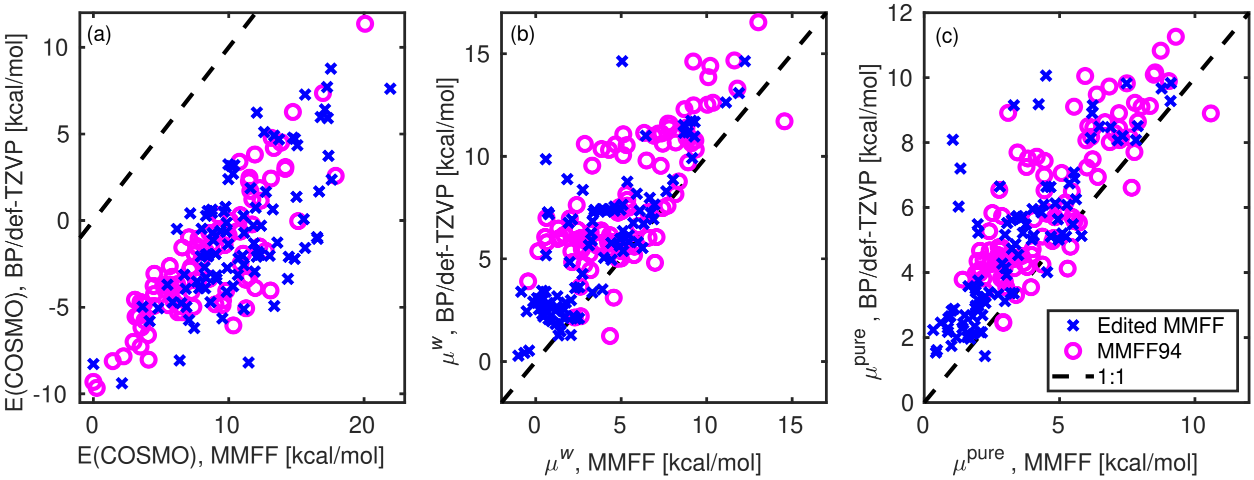

Figure S2: (a) COSMO energies and chemical potentials (μ) in (b) water and (c) pure compound of geometries optimized at the MMFF and BP/def-TZVP levels of theory. The COSMO energies (E(COSMO)) are given relative to the lowest COSMO energy conformer after MMFF optimization.

It is more likely that new intramolecular hydrogen bonds are formed in geometry optimization at the BP/def-TZVP level of theory, rather than existing hydrogen bonds breaking. The geometry optimization therefore generally increases the chemical potential in water and pure compound. This is optimal for applications, where the goal is to find lowest chemical potential conformers, because taking a subset of the MMFF optimized conformers based on their chemical potentials is unlikely to discard any lowest chemical potential conformers from further calculations. Comparing the two MMFF force fields reveals that for some conformers, BP/def-TZVP geometry optimization decreases the chemical potential compared to the MMFF94 optimized geometry. In this small sample, we were also able to find more low chemical potential conformers using the edited MMFF force field than the original MMFF94 force field. This difference is likely caused by the peroxy acid group in the molecule. The edited MMFF leads to around 80 % of the conformers having C-O-O-H dihedral angle of the peroxy acid group at 180° (no intramolecular H-bond and higher intermolecular interaction), as opposed to the 0° dihedral angles dominating the conformer sampling using the MMFF94 force field.

We additionally tested the edited MMFF force field for a set of 200 molecules using a

single conformer of each molecule. Figure S3 shows how the COSMO energy and chemical potentials change between the edited MMFF and BP/def-TZVP geometries. This comparison shows how the two force fields work for molecules that contain fewer functional groups and only 3 of them contain peroxy acid groups. We can observe a similar trend, where the chemical potentials are similar or higher after the geometry optimization at the BP/def-TZVP level of theory. It should be noted that the conformers were created separately with both of the MMFF force fields, which led to different conformers for most of the molecules.

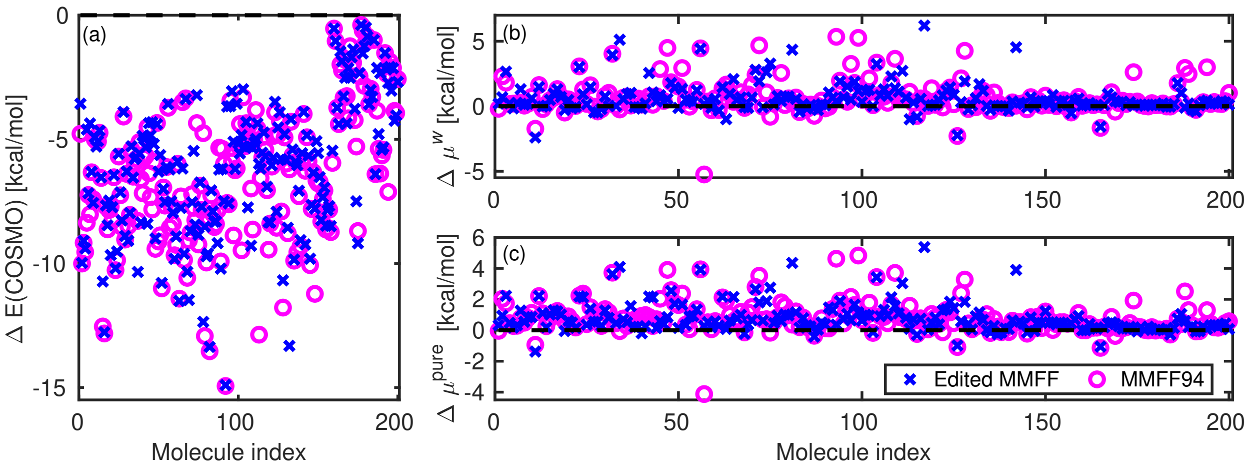

Figure S3: Difference in (a) COSMO energies and chemical potentials (μ) in (b) water and (c) pure compound of geometries optimized at the MMFF and BP/def-TZVP levels of theory.

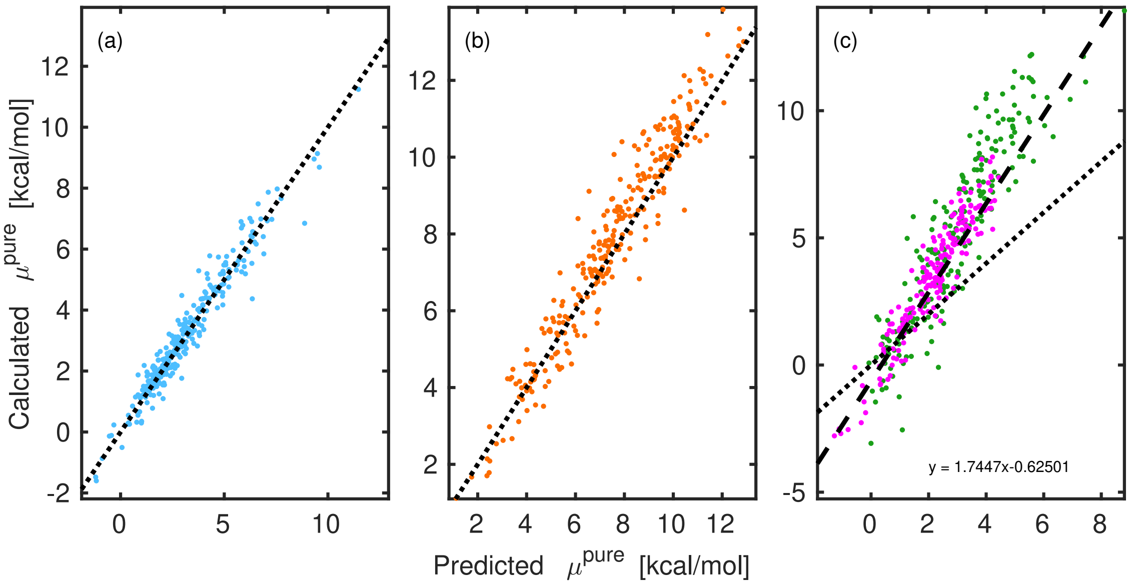

Figure S4: Predicted and calculated chemical potentials (μ^{pure}) of (a) test1, (b) test2 and (c) test3 in pure compound. Only a small subset of the test data points, taken at constant intervals, are shown in the figures for clarity. In (c), the dashed line is a linear fit to all of the molecules in test3. The green and magenta points are for the same isomers as in Figure 3 of the main article.

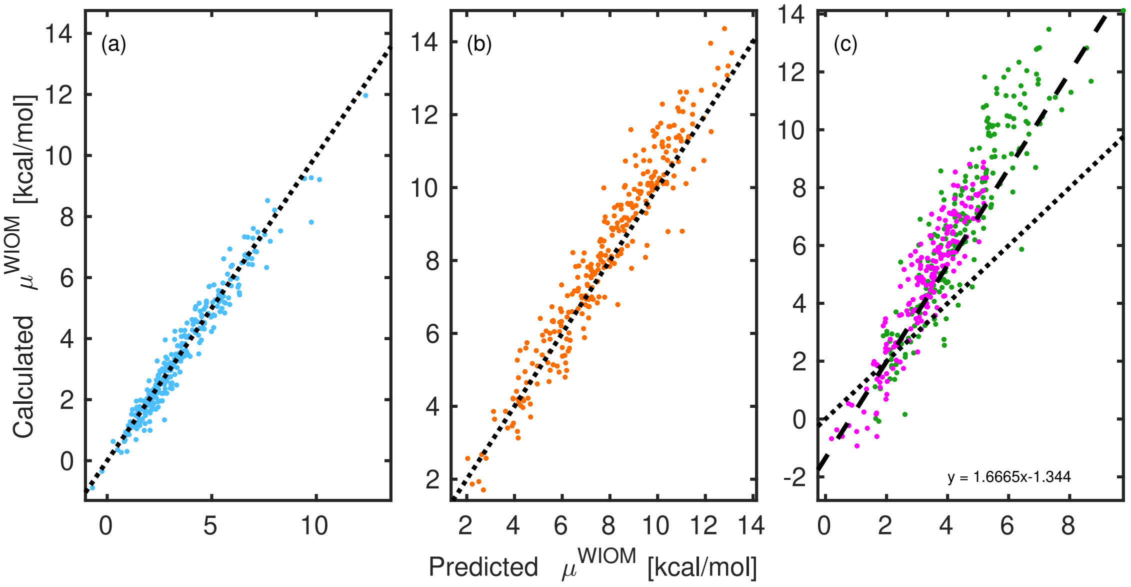

Figure S5: Predicted and calculated chemical potentials (μ^{WIOM}) of (a) test1, (b) test2 and (c) test3 in infinite dilution in WIOM. Only a small subset of the test data points, taken at constant intervals, are shown in the figures for clarity. In (c), the dashed line is a linear fit to all of the molecules in test3. The green and magenta points are for the same isomers as in Figure 3 of the main article.

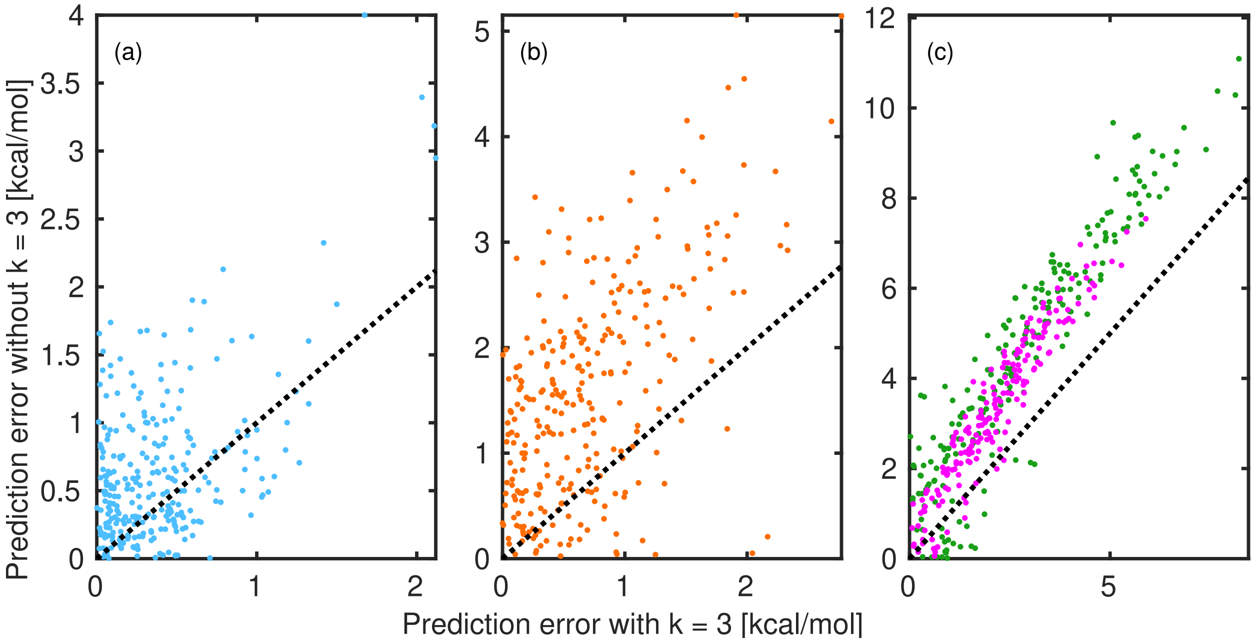

Figure S6: The absolute errors in predicted chemical potentials of (a) test1, (b) test2 and (c) test3 in water solvent with and without the $k = 3$ tensors in the descriptor. Only a small subset of the test data points, taken at constant intervals, are shown in the figures for clarity. The green and magenta points are for the same isomers as in Figure 3 of the main article.

S2 Pseudo-Chemical Potential

Instead of the conventional chemical potential, COSMO*therm* utilises the pseudo-chemical potential.[S5] Pseudo-chemical potential μ_i^* is calculated in COSMO*therm* using the screening charge densities σ:

$$\mu_i^* = \mu_i^{C,S} + \int p_i(\sigma)\mu_S(\sigma)d\sigma, \tag{S1}$$

where $p_i(\sigma)$ is the un-normalized σ-profile, and $\mu_S(\sigma)$ is the chemical potential of a surface segment with the screening charge density σ, which describes the affinity of the solution S to a surface of screening charge density σ, and $\mu_i^{C,S}$ is the combinatorial contribution to the chemical potential.

The conventional chemical potential (μ_i) in a solution S is defined with respect to the chemical potential in a given reference state μ_i° with constant temperature T and pressure P as

$$\mu_i^S = \mu_i^\circ(T, P) + RT \ln a_i, \tag{S2}$$

where R is the gas constant and $a_i = a_i^S$ is the activity of component i in a given solution, with respect to the chosen reference state. The pseudo-chemical potential μ_i^* is defined as

$$\mu_i^{*,S} = \mu_i^\circ(T, P) + RT \ln \gamma_i, \tag{S3}$$

where γ_i is the activity coefficient of component i in solution S. By definition, when component i is in the reference state, the activity coefficient of component i is 1. This means that in the reference state:

$$\mu_i^{*\circ}(T, P) = \mu_i^\circ(T, P) \tag{S4}$$

For simplicity, we have used μ to represent pseudo-chemical potential (defined here as μ^*) in the article.

S3 ML Model Extrapolation

Here, we present test calculations on extrapolating the model to larger molecule sizes. All of the following calculations were done using chemical potentials in the infinite dilution in water and geometries optimized at the MMFF level of theory (train1 and test1).

To test how well EMLM is able to predict chemical potentials of molecules larger than those included in the training data, we trained the EMLM model with only the smaller molecules of train1. First, we trained the model with molecules that contain 3-10 non-hydrogen atoms (10250 conformers in total). The molecules of this data set contain at most 9 carbon atoms and 6 oxygen atoms. We trained the model using both 25 % and 100 % of the total number of conformers as reference points. No significant difference was seen between the number of reference points, so we used 25 % of the training data as reference points in the following calculations.

We then predicted chemical potentials of the larger molecules containing 11-19 non-hydrogen atoms. Figure S7 (red circles) shows the RMSE between the calculated and predicted chemical potentials as a function of molecule size. With the linear fit scaling similar to what was done for test3, the RMSE increases from 0.49 kcal/mol for molecules with 11 non-hydrogen atoms to 0.85 kcal/mol for molecules with 14 non-hydrogen atoms. There are some outliers already in the predicted chemical potentials of the 15 non-hydrogen atom molecules (maximum 10 carbon atoms and 8 oxygen atoms) and the prediction accuracy quickly decreases when more atoms are added to the molecules. Looking at separate molecules in the test data set of 19 non-hydrogen atom molecules shows that the prediction is very accurate for some molecules (RMSE = 0.9 kcal/mol) and very poor for others (RMSE = 3.8 kcal/mol).

We also trained one model with atoms containing 3-14 non-hydrogen atoms (53350 conformers in total, shown in blue crosses in Figure S7) and the results are similar to the model trained with molecule containing 3-10 non-hydrogen atoms. Both models work well (RMSE $<$ 1 kcal/mol) for molecules containing up to 4 non-hydrogen atoms more than the molecules in the training data.

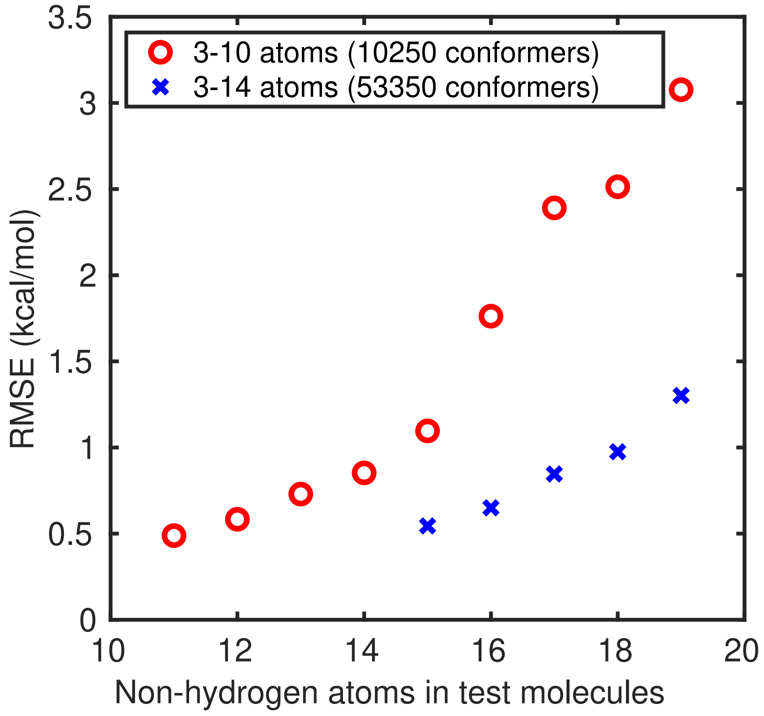

Figure S7: Root mean square errors (RMSE) for test molecules that are outside the size range (in terms of non-hydrogen atoms) of the training data. The size of test molecules is given on the x-axis and results of models containing different molecule sizes are shown with different markers.

Next, we tested how many molecules containing 19 non-hydrogen atoms (50 conformers each) need to be added to the training data that consisted of 3-10 non-hydrogen atom molecules, in order to accurately predict chemical potentials of molecules containing 19 non-hydrogen atoms. The additional molecules to the training data were selected from test1, in order to use the same test molecules as in the test calculations above. From Figure S8 we can see that the addition of 2 molecules with 19 non-hydrogen atoms decreases the RMSE (linear fit scaling) from 3.1 kcal/mol to 1.9 kcal/mol. When 10 or more molecules are added to the training data, no outliers are seen in the predicted chemical potentials of the test molecules. Adding even more molecules containing 19 non-hydrogen atoms to the training data does not lead to significant improvements to the prediction. Similar RMSE was obtained by including different sets of 10 molecules from test1 to the training data.

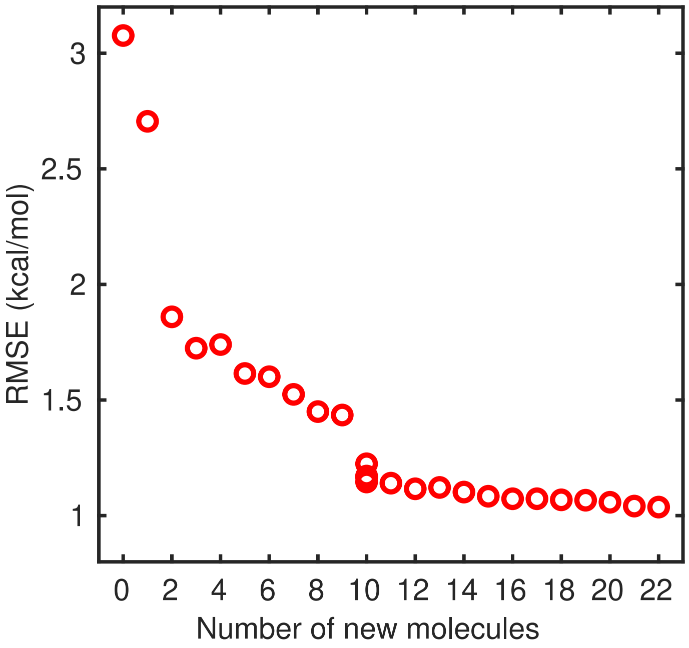

Figure S8: RMSE (scaled to linear fit) of molecules containing 19 non-hydrogen atoms, when the original training data consist of molecules containing 3-10 non-hydrogen atoms and new molecules, also containing 19 non-hydrogen atoms, are added to the training data. The result for 10 additional 19 non-hydrogen atom molecules was confirmed with 4 different sets of molecules.

S4 Many-Body Tensor Representation

Many-Body Tensor Representation (MBTR)[S6] is a global descriptor for atomic structures, therefore it encodes a full structure into a single representation. In this study we used the implementation published in DScribe package version 0.4.1.[S7] There MBTR has three different levels or measured properties: atomic number, inverse of the Euclidean distances between atoms (or sometimes just Euclidean distance) and the cosines of the angles formed by the sets of three atoms (or sometimes just angles).

The first type of MBTR (atom numbers) is calculated as

$$\mathrm{MBTR}_1^{Z_i}(x) = \sum_{l}^{|Z_i|} \frac{1}{\sigma_1\sqrt{2\pi}} \exp\left(\frac{(x-Z_l)^2}{2\sigma_1^2}\right), \tag{S5}$$

where the summation goes over all atoms with atomic number Z_i. The parameter σ_1 determines the amount of broadening and x is as sweeping variable probing the values from the function. Effectively, this scheme counts the number of atoms with certain atomic number.

The second type of MBTR (distances) is calculated as

$$\mathrm{MBTR}_2^{Z_i,Z_j}(x) = \sum_{l}^{|Z_i|}\sum_{m}^{|Z_j|} w_2(\mathbf{r}_l,\mathbf{r}_m) \frac{1}{\sigma_2\sqrt{2\pi}} \exp\left(\frac{(x-g_2(\mathbf{r}_l,\mathbf{r}_m))^2}{2\sigma_2^2}\right), \tag{S6}$$

where $g_2(\mathbf{r}_l,\mathbf{r}_m) = 1/|\mathbf{r}_l-\mathbf{r}_m|$ is an inverse distance between the positions of atom l and m. Weighting is done exponentially as $w_2(\mathbf{r}_l,\mathbf{r}_m) = \exp(-s_2|\mathbf{r}_l-\mathbf{r}_m|)$. Here, σ_2 and s_2 are parameters determining the broadening and the steepness of the weighting. The summations go over atoms with atomic numbers of Z_i and Z_j.

The third type of MBTR (angles) is calculated as

$$\mathrm{MBTR}_3^{Z_i,Z_j,Z_k}(x) = \sum_{l}^{|Z_i|}\sum_{m}^{|Z_j|}\sum_{n}^{|Z_k|} w_3(\mathbf{r}_l,\mathbf{r}_m,\mathbf{r}_n) \frac{1}{\sigma_3\sqrt{2\pi}} \exp\left(\frac{(x-g_3(\mathbf{r}_l,\mathbf{r}_m,\mathbf{r}_n))^2}{2\sigma_3^2}\right), \tag{S7}$$

where $g_3(\mathbf{r}_l,\mathbf{r}_m,\mathbf{r}_n) = \cos(\angle(\mathbf{r}_l-\mathbf{r}_m,\mathbf{r}_n-\mathbf{r}_m))$, i.e., the cosine of the angle formed by atoms

l, m and n centered on atom m. The weighting is $w_3(\mathbf{r}_l, \mathbf{r}_m, \mathbf{r}_n) = \exp(-s_3(|\mathbf{r}_l - \mathbf{r}_m| + |\mathbf{r}_m - \mathbf{r}_n| + |\mathbf{r}_l - \mathbf{r}_n|))$. The parameters are similar to the two previous versions described above.

MBTR has several parameters, part of which are seen in equations (S5), (S6) and (S7). The parameters for every level of MBTR are broadening σ_k, weighting s_k (excluding the atomic number), the minimum value of the sweeping variable x, the maximum value of the sweeping variable x, the number of points sampled with x between minimum and maximum values and a threshold value for the summation. The threshold determines the minimum value of the description function included into the summation. This is used to exclude effects from faraway atoms with small weights. The used parameters are listed in the Table S1.

Table S1: Optimized MBTR Parameters According to the Level of Description.

MBTR_k	σ_k	s_k	x_{min}	x_{max}	N_x	threshold
1	0.04	-	0	10	100	-
2	0.025	0.5	0	1.25	100	10^{-3}
3	0.12	0.8	-1	1	100	10^{-3}

S5 Extreme Minimal Learning Machine

Extreme Minimal Learning Machine (EMLM) is a kernel-based ML method that uses Euclidean distances as similarity measure. The training requires input data $\mathbf{X} = \{\mathbf{x}_i\}_{i=1}^{N} \in \mathbb{R}^{N\times n_x}$ and corresponding output data $\mathbf{Y} = \{\mathbf{y}_i\}_{i=1}^{N} \in \mathbb{R}^{N\times n_y}$. From $\mathbf{X}$ we sample K reference points forming a reference data set $\mathbf{Q} = \{\mathbf{q}_j\}_{j=1}^{K} \in \mathbb{R}^{K\times n_x}$. The actual training is done via solving a regularized least-squares optimization problem[S8]

$$\min_{\mathbf{W}\in\mathbb{R}^{K\times n_y}} J(\mathbf{W}) = \frac{1}{2N}\sum_{i=1}^{N}\left|\mathbf{d}_i^T\mathbf{W} - \mathbf{y}_i^T\right|^2 + \frac{\beta}{2K}\sum_{i=1}^{K}\sum_{j=1}^{n_y}|W_{ij}|^2. \tag{S8}$$

Here $\mathbf{W} \in \mathbb{R}^{K\times n_y}$ is a weight matrix of the EMLM model. The vector $\mathbf{d}_i \in \mathbb{R}^K$ contains Euclidean distances between the ith input data point and all reference points in $\mathbf{Q}$. The amount of regularization is adjusted with parameter β. Usually, β is set close to zero, when regularization is not needed to prevent over fitting. In this study, it was set to 10^{-9}.

The minimum of the equation S8 can be found by writing the equation in a matrix form and finding the zero point of the first derivative.

$$\frac{1}{N}\mathbf{D}^T\left(\mathbf{D}\mathbf{W} - \mathbf{Y}\right) + \frac{\beta}{K}\mathbf{W} = 0 \tag{S9}$$

$$\left(\mathbf{D}^T\mathbf{D} + \beta\frac{N}{K}\mathbf{I}\right)\mathbf{W} = \mathbf{D}^T\mathbf{Y} \tag{S10}$$

Matrix $\mathbf{D} \in \mathbb{R}^{N\times K}$ contains all Euclidean distances between training data points and references. After solving the weights, new output could be predicted by calculating Euclidean distances between given input and saved reference points forming distance vector $\mathbf{d} \in \mathbb{R}^K$, and then computing the multiplication $\mathbf{d}^T\mathbf{W}$. In addition to the regularization parameter β, EMLM has just a single hyper parameter, which is the number of reference points. This is a very useful feature, because MBTR has several parameters as shown in the Table S1.

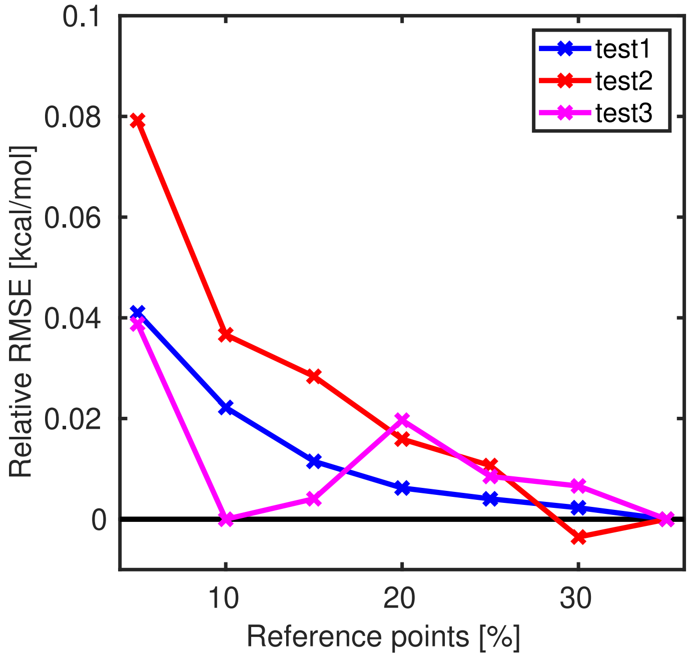

Figure S9: Convergence of the prediction of chemical potential in water as a function of reference points used in the training of the EMLM model.The RMSE are given relative to the value of 35 % reference points.

References

(S1) Hyttinen, N.; Prisle, N. L. Improving Solubility and Activity Estimates of Multifunctional Atmospheric Organics by Selecting Conformers in COSMO*therm*. *J. Phys. Chem. A* **2020**, *124*, 4801–4812.

(S2) Vainio, M. J.; Johnson, M. S. Generating Conformer Ensembles Using a Multiobjective Genetic Algorithm. *J. Chem. Inf. Model.* **2007**, *47*, 2462–2474.

(S3) TURBOMOLE, V7.4.1, a development of University of Karlsruhe and Forschungszentrum Karlsruhe GmbH, 1989-2007, TURBOMOLE GmbH, since 2007. 2019.

(S4) BIOVIA COSMO*therm*, *Release 2021*; Dassault Systèmes, 2021.

(S5) Ben-Naim, A. *Solvation Thermodynamics*; Plenum Press: New York and London, 1987.

(S6) Huo, H.; Rupp, M. Unified Representation of Molecules and Crystals for Machine Learning. *arXiv* **2017**, 1704.06439v3 [physics.chem–ph].

(S7) Himanen, L.; Jäger, M. O.; Morooka, E. V.; Federici Canova, F.; Ranawat, Y. S.; Gao, D. Z.; Rinke, P.; Foster, A. S. DScribe: Library of Descriptors for Machine Learning in Materials Science. *Comput. Phys. Commun.* **2020**, *247*, 106949.

(S8) Kärkkäinen, T. Extreme Minimal Learning Machine: Ridge Regression with Distance-Based Basis. *Neurocomputing* **2019**, *342*, 33–48.
